# Glasgow Prognostic Score predicts chemotherapy‐triggered acute exacerbation‐interstitial lung disease in patients with non‐small cell lung cancer

**DOI:** 10.1111/1759-7714.13792

**Published:** 2021-01-21

**Authors:** Ryota Kikuchi, Hiroyuki Takoi, Takao Tsuji, Yoko Nagatomo, Akane Tanaka, Hayato Kinoshita, Mariko Ono, Mayuko Ishiwari, Kazutoshi Toriyama, Yuta Kono, Yuki Togashi, Kazuhiro Yamaguchi, Akinobu Yoshimura, Shinji Abe

**Affiliations:** ^1^ Department of Respiratory Medicine Tokyo Medical University Hospital Tokyo Japan; ^2^ Respiratory Center Otsuki Municipal Central Hospital Yamanashi Japan; ^3^ Department of Clinical Oncology Tokyo Medical University Hospital Tokyo Japan

**Keywords:** Acute exacerbation, Glasgow Prognostic Score, interstitial lung disease, non‐small cell lung cancer, prognosis

## Abstract

**Background:**

Interstitial lung disease (ILD) in patients with non‐small cell lung cancer (NSCLC) worsens the prognosis for overall survival (OS) due to chemotherapy‐triggered acute exacerbation (AE)‐ILD. The Glasgow Prognostic Score (GPS), which is based on serum C‐reactive protein and albumin levels, has been suggested as a reliable prognostic tool for mortality in cancer patients, including NSCLC. In this study, we investigated whether GPS is a predictor for chemotherapy‐triggered AE‐ILD and the prognosis in patients with NSCLC and pre‐existing ILD.

**Methods:**

We conducted a retrospective review on 56 NSCLC and ILD patients at our hospital who received platinum agent‐based treatment as first‐line chemotherapy between June 2010 and May 2019. We categorized these patients according to their GPS (0–2) and compared the incidence of chemotherapy‐triggered AE‐ILD and OS.

**Results:**

The GPS 0, 1, and 2 groups included 31, 16, and nine patients, respectively, out of 56. A total of 12 (21.4%) patients showed chemotherapy‐triggered AE‐ILD. The median OS was at 11.5 months (95% confidence interval: 8.0–15.1). The incidence of chemotherapy‐triggered AE‐ILD within the first year of chemotherapy in the GPS 0, 1, and 2 groups was three (9.6%), four (25.0%), and five (55.5%), and the median OS time was 16.9, 9.8 and 7.6 months, respectively. Univariate and multivariate analyses indicated that only GPS 2 could predict both chemotherapy‐triggered AE‐ILD and OS (*P* < 0.05).

**Conclusions:**

GPS assessment of patients with NSCLC and pre‐existing ILD is a valuable prognostic tool for predicting chemotherapy‐triggered AE‐ILD and OS.

**Key points:**

**Significant findings of the study:**

We found that GPS 2 was an independent risk factor for chemotherapy‐triggered AE‐ILD and prognosis in patients with ILD associated with NSCLC.

**What this study adds:**

GPS may potentially enable the discrimination of patients tolerant of chemotherapy from those at an increased risk of AE‐ILD and predict the prognosis in patients with NSCLC and ILD receiving chemotherapy.

## Graphical abstract

We assessed GPS and AE‐ILD/prognosis in patients with NSCLC and ILD.

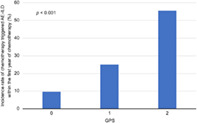

## Introduction

Interstitial lung disease (ILD), including idiopathic pulmonary fibrosis (IPF), is concomitant in 10%–30% of patients with lung cancer.^1^, ^2^, ^3^, ^4^, ^5^, ^6^, ^7^ The presence of ILD diminishes the prognosis of lung cancer patients,^8^, ^9^, ^10^ with one study on non‐small cell lung cancer (NSCLC) showing the 12–14 month overall survival (OS) to be shortened by two to five months in ILD patients.^11^ Chemotherapy has been previously reported to acutely exacerbate ILD (AE‐ILD) in 10%–30% of cases with a mortality rate of 22%–27%.^12^, ^13^, ^14^, ^15^, ^16^ Improving the prognosis of patients with lung cancer and pre‐existing ILD depends on identifying the risk factors for chemotherapy‐triggered AE‐ILD.

A recent meta‐analysis of lung cancer pharmacotherapy showed that ILD has increased the cause of treatment‐related deaths.^17^ However, clinical trials on lung cancer often exclude patients with ILD because of chemotherapy‐triggered AE‐ILD.^18^ Consequently, few studies have focused on patients with lung cancer and concomitant ILD.

The inflammation‐based Glasgow Prognostic Score (GPS), is calculated by the C‐reactive protein (CRP) and albumin (Alb) levels and serves as a reliable long‐term prognostic indicator of solid cancers, including lung cancers; a higher GPS score denotes a worse prognosis.^19^, ^20^, ^21^, ^22^, ^23^ Gioulbasanis *et al*. ^24^ used the GPS to show toxicity induced by platinum‐based chemotherapy in lung cancer patients. Kang *et al*. ^25^ showed the GPS to be a useful prognostic indicator for acute exacerbation of idiopathic pulmonary fibrosis (AE‐IPF), which accounts for over 50% of idiopathic ILD cases. To date, no reports have examined the relationship between GPS and lung cancer with concomitant ILD in patients undergoing chemotherapy. Therefore, we decided to investigate whether the GPS could be used as a prognostic indicator or a predictor of chemotherapy‐triggered AE‐ILD for lung cancer patients with concomitant ILD.

## Methods

### Patients

The ethical committee of Tokyo Medical University approved this retrospective study protocol (approval number: T2020‐0096) and waived the requirement for informed consent, although patients could choose not to participate. All methods were performed in accordance with relevant guidelines/regulations. We analyzed the medical records of consecutive NSCLC and ILD patients receiving chemotherapy at the Tokyo Medical University Hospital between June 2010 and May 2019. The study excluded concerned patients who did not receive chemotherapy, were transferred to other medical centers before chemotherapy, were diagnosed with cancer accompanied by obstructive pneumonia, or had clinical evidence of infections. Physicians diagnosed the metastasis stage and tumor nodes according to the Tumor, Node, Metastasis classification (eighth edition).^26^ Individualized chemotherapy regimens were developed for patients based on drug effectiveness, risk of AE‐ILD, and patient needs. We excluded patients using chemotherapeutic drugs either contraindicated or avoided in Japan for patients with ILD (i.e., epidermal growth factor receptor tyrosine kinase inhibitor and gemcitabine). No patient had undergone chest radiotherapy.

The GPS scores of patients were determined the day before initializing first‐line chemotherapy treatment and were based on serum CRP and Alb levels. Patients were classified into one of the three groups based on their GPS designation: GPS 0: CRP ≦10 mg/L and Alb ≧35 g/L; GPS 1: CRP >10 mg/L or Alb <35 g/L; and GPS 2: CRP >10 mg/L; and Alb <35 g/L.^19^, ^20^ Patients whose CRP or Alb levels were not measured were excluded from the study. Outcomes were compared among the three groups by the incidence of chemotherapy‐triggered AE‐ILD and OS. The definition of OS was the time from the start of first‐line chemotherapy administration to death or at censoring. Patients who lived beyond 31 May 2019 were censored.

Two pulmonologists (R.K and T.T) independently evaluated high‐resolution computed tomography (HRCT) chest images acquired before the administration of first‐line chemotherapy without knowledge of patient outcomes. ILD diagnosis was determined by findings of reticulation shadow, ground‐glass attenuation (GGA), and/or honeycombing in both lung fields. Computer tomography (CT) findings of ILD were categorized into one of two groups according to the recent guidelines for IPF^27^: (i) Usual interstitial pneumonia (UIP) manifestations; and (ii) non‐UIP manifestations. In the non‐UIP group, we included a probable UIP pattern, an indeterminate UIP pattern, and a pattern with an alternative diagnosis. Disagreements in the HRCT findings were resolved by discussion and consensus. We excluded secondary ILD (ie, interstitial pneumonia associated with connective tissue disease or antineutrophil cytoplasmic antibody‐associated ILD) because these diseases affect elevated CRP levels and/or hypoalbuminemia. No patient received immunosuppressive therapy before chemotherapy.

AE‐ILD included all acute respiratory events with newly appearing, bilateral ground‐glass opacification (GGO) and/or interstitial shadows that could not be explained by infectious disease, cardiac failure, or fluid overload.^28^, ^29^, ^30^, ^31^, ^32^ AE‐ILD triggered by chemotherapy was described as the onset of AE within four weeks of the last administration of chemotherapy.^29^, ^30^, ^31^, ^32^

### Statistical analysis

The data were described either as percentages or medians (range). Kruskal–Wallis and chi‐square tests were used for comparison of baseline patient characteristics. Logistic regression univariate and multivariate analyses determined the chemotherapy‐triggered AE‐ILD incidence rate. Independent risk factors for survival were determined by the Cox proportional hazards regression model using univariate and multivariate analyses. A Kaplan–Meier analysis estimated median survival, and a log‐rank test determined patient survival within the GPS groups 0–2. *P*‐values < 0.05 were considered statistically significant. Statistical analyses were performed using SPSS software version 26.0 (IBM Corp., Armonk, NY, US).

## Results

### Patient characteristics

A total of 74 patients with NSCLC and concomitant ILD were admitted to our hospital during the study period. A total of 18 patients were excluded because of secondary ILD of known etiology, lack of measurement of CRP or Alb, not using platinum‐based chemotherapy as first‐line treatment, no chemotherapy, transferring to another hospital before chemotherapy, infectious condition, administering gemcitabine, or using erlotinib. We analyzed data from 56 patients as summarized in Fig S1.

Fifty‐six patients diagnosed with NSCLC and pre‐existing ILD were treated with chemotherapy, and their baseline and clinicopathological characteristics are summarized in Table 1. The median age of patients was 71 years; 40 (71.4%) patients were male and 53 (94.6%) had a smoking history. Forty‐nine patients (87.5%) had an Eastern Cooperative Oncology Group Performance Status (PS) of either 0 or 1 and 28 (50.0%) had adenocarcinoma. Under the Tumor, Node, Metastasis lung cancer staging system, 46 (82.1%) patients showed stage IV NSCLC or recurrent disease after surgical resection. Based on the HRCT findings of ILD, 25 (44.6%) patients had a UIP pattern, while the remaining showed a non‐UIP pattern.

**Table 1:** Baseline characteristics of all patients and clinical manifestation according to the GPS

	Total	GPS 0	GPS 1	GPS 2	*P*‐value
No. of patients	56	31	16	9	0.732
Age (years)	71	71	72	71	
Median range		66–76	68–77	65–74	
Sex (%)					
Male	40 (71.4)	24 (77.4)	12 (75.0)	4 (44.4)	0.145
Female	16 (28.6)	7 (22.6)	4 (25.0)	5 (55.6)	
Performance status (%)					0.303
0, 1	49 (87.5)	29 (93.5)	13 (81.3)	7 (77.8)	
2–4	7 (12.5)	2 (6.5)	3 (18.8)	2 (22.2)	
Smoking status (%)					0.790
Never smoked	3 (5.4)	2 (6.5)	0 (0.0)	1 (11.1)	
Former smoker	41 (73.2)	23 (74.2)	12 (75.0)	6 (66.7)	
Current smoker	12 (21.4)	6 (19.4)	4 (25.0)	2 (22.2)	
Pack‐years	50.0	50.0	49.5	53.0	0.702
Median range		36.7–82.5	38.1–58.7	15.0–60.2	
BMI (kg/m^2^)	23.1	23.9	22.8	20.8	0.089
Median range		19.6–25.7	21.5–23.8	19.8–22.4	
Histology (%)					0.305
Adenocarcinoma	28 (50.0)	17 (54.8)	5 (31.3)	6 (66.7)	
Squamous cell carcinoma	25 (44.6)	13 (41.9)	9 (56.3)	3 (33.3)	
Clinical stage (%)					0.788
III	10 (17.9)	6 (19.4)	2 (12.5)	2 (22.2)	
IV or recurrence	46 (82.1)	25 (80.6)	14 (87.5)	7 (77.8)	
WBC (/μL)	7100	6400	8400	7700	0.034
Median range		5500–7500	5825–9750	7350–9100	
Neutrophils (/μL)	4436	4015	6085	5035	0.018
Median range		3478–4831	4065–7407	3033–6781	
Lymphocytes (/μL)	1505	1650	1413	1243	0.122
Median range		1232–2056	970–1731	535–1974	
Eosinophils (/μL)	137	159	129	100	0.845
Median range		83–258	51–251	37–242	
KL‐6 (U/mL)	624	591	600	1034	0.301
Median range		462–843	433–961	564–1480	
%predicted FVC (%)	103.1	104.6	98.5	84.3	0.612
Median range		95.0–111.0	79.8–126.5	55.8–121.3	
HRCT pattern (%)					0.331
UIP pattern	25 (44.6)	12 (38.7)	7 (43.7)	6 (66.7)	
Non‐UIP pattern	31 (55.4)	19 (61.3)	9 (56.2)	3 (33.3)	
Emphysema (%)					0.752
Yes	45 (80.4)	26 (83.9)	12 (75.0)	7 (77.8)	
No	11 (19.6)	5 (16.1)	4 (25.0)	2 (22.2)	

BMI, body mass index; FVC, forced vital capacity; GPS, Glasgow Prognostic Score; HRCT, high‐resolution computed tomography; KL‐6, Krebs von den Lungen‐6; UIP, usual interstitial pneumonia; WBC, white blood cell.

### Correlation of the GPS with clinicopathological parameters

The number of patients in each of the three groups was GPS 0 (*n* = 31), GPS 1 (*n* = 16), and GPS 2 (*n* = 9) (Table 1). Significant differences in clinical characteristics were found in white blood cell (WBC) levels (6400/μL, 8400/μL, and 7700/μL, respectively; *P* < 0.05) and neutrophil levels (4015/nL, 6085/nL, and 5035/nL; *P* < 0.05) (Table 1).

### Chemotherapy‐triggered AE‐ILD

**Figure 1:**
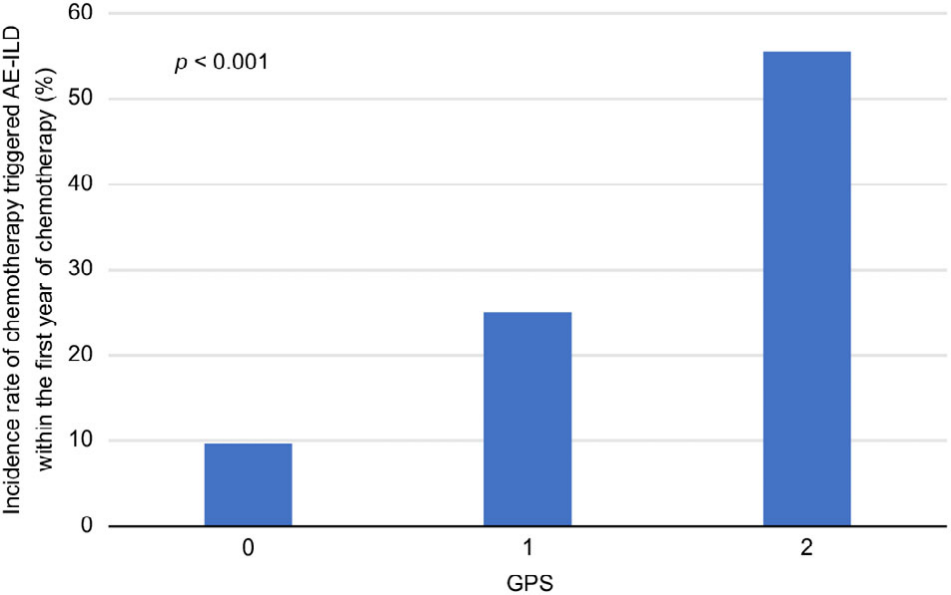
Overall incidence rate of chemotherapy‐triggered AE‐ILD according to the GPS (GPS 0, *n* = 31; GPS 1, *n* = 16; GPS 2, *n* = 9). AE, acute exacerbation; GPS, Glasgow Prognostic Score; ILD, interstitial lung disease. [Correction added on 9 February 2021, after first online publication: the values in the horizontal axis of figure 1 have been corrected from ‘1, 2, and 3’ to ‘0, 1, and 2’.]

A total of 12 of the 56 patients (21.4%) experienced chemotherapy‐triggered AE‐ILD during the study. The incidence of chemotherapy‐triggered AE‐ILD within the first year of chemotherapy were three (9.6%), four (25.0%), and five (55.5%) in the GPS groups of 0, 1, and 2, respectively (Fig 1), which suggested that the GPS is associated with the incidence rate of AE‐ILD (*P* < 0.001).

We determined the risk factors for chemotherapy‐triggered AE‐ILD using univariate and multivariate analyses of each variable (Table 2). Univariate analysis showed that %predicted forced vital capacity (FVC) and GPS 2 were significantly associated with risk factors of AE‐ILD (odds ratio [OR] of %predicted FVC: 0.96, 95% confidence interval CI: 0.92–0.99: *P* = 0.028; OR of GPS 2: 11.66, 95% CI: 1.98–68.75, *P* = 0.007). A GPS score of 2 was the only predictor that exhibited a significant association with the incidence of chemotherapy‐triggered AE‐ILD, as revealed by multivariate analysis (OR of GPS 2: 8.86, 95% CI: 1.05–74.45, *P* = 0.044).

**Table 2:** Univariate and multivariate analyses of chemotherapy‐triggered AE‐ILD in patients with lung cancer and ILD

	Univariate analysis			Multivariate analysis		
Variable	OR	95% CI	*P‐*value	OR	95% CI	*P‐*value
Age, per year increment	0.98	0.89–1.07	0.693			
Sex (male vs. female)	0.46	0.12–1.77	0.260			
Performance status (0, 1 vs. 2–4)	1.56	0.26–9.25	0.625			
Smoking status (never vs. current or former)	0.52	0.043–6.32	0.611			
Pack‐years, per pack year increment	0.99	0.97–1.01	0.547			
BMI, per kg/m^2^ increment	0.92	0.75–1.14	0.474			
Histology (adenocarcinoma vs. nonadenocarcinoma)	1.00	0.27–3.58	1.000			
Clinical stage (III vs. IV or recurrence)	2.82	0.32–24.8	0.349			
WBC, per/μL increment	1.00	1.00–1.00	0.141			
Neutrophils, per/μL increment	1.00	1.00–1.00	0.083			
Lymphocytes, per/μL increment	0.99	0.99–1.00	0.113			
Eosinophils, per/μL increment	0.99	0.99–1.00	0.256			
KL‐6, per U/mL increment	1.00	0.99–1.00	0.696			
HRCT pattern (UIP pattern vs. non‐UIP pattern)	0.90	0.26–3.07	0.877			
Emphysema (yes vs. no)	0.66	0.14–3.03	0.600			
%predicted FVC, per % increment	0.96	0.92–0.99	0.028	0.97	0.93–1.00	0.110
GPS						
0	1 (Ref)			1 (Ref)		
1	3.11	0.60–16.08	0.176	3.86	0.52–28.54	0.185
2	11.66	1.98–68.75	0.007	8.86	1.05–74.45	0.044

AE, acute exacerbation; BMI, body mass index; CI, confidence interval; FVC, forced vital capacity; GPS, Glasgow Prognostic Score; HRCT, high‐resolution computed tomography; ILD, interstitial lung disease; KL‐6, Krebs von den Lungen‐6; OR, odds ratio; Ref, reference; UIP, usual interstitial pneumonia; WBC, white blood cells.

The chemotherapy regimens are shown in Table S1. The first‐line chemotherapy treatment most often combined carboplatin and paclitaxel with/without bevacizumab, while docetaxel (DOC) was the most used second‐line chemotherapy option. Eight patients (32%) who received DOC presented AE‐ILD, which was a percentage higher than any other chemotherapy drug (Table S2).

### Prognosis

**Figure 2:**
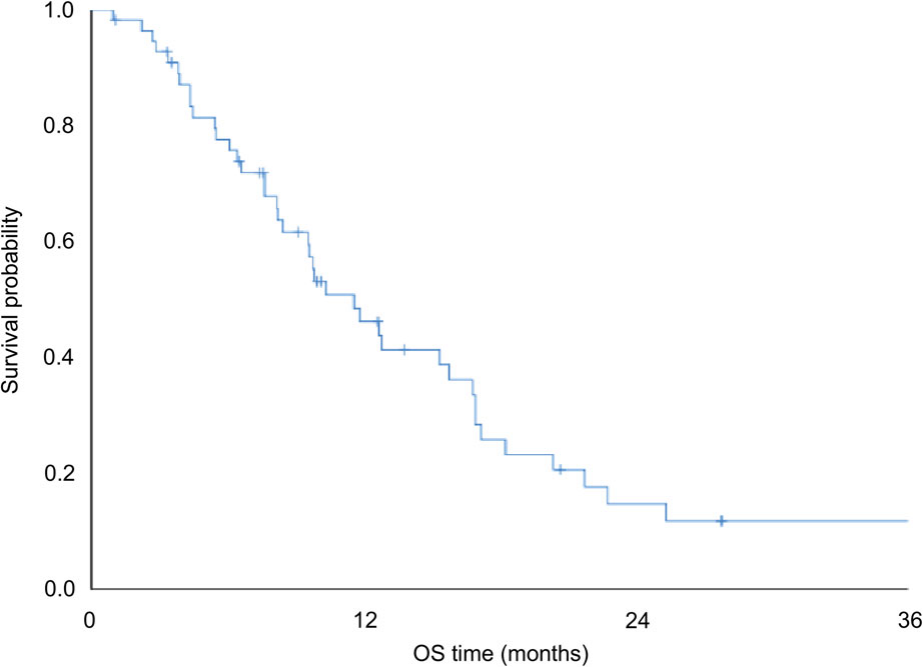
OS curve of patients with ILD associated with NSCLC who received chemotherapy (*n* = 56). ILD, interstitial lung disease; NSCLC, non‐small cell lung cancer; OS, overall survival Median OS (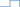)Total cohort 11.5 months (*n* = 56).

**Figure 3:**
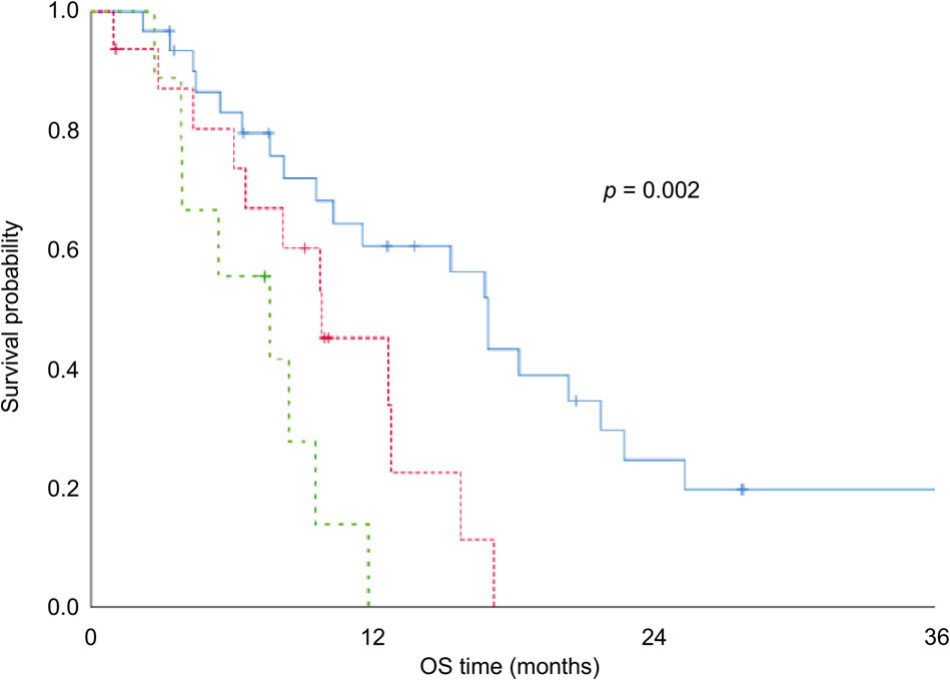
OS curve of patients with ILD associated with NSCLC who received chemotherapy according to the GPS (GPS 0, *n* = 31; GPS 1, *n* = 16; GPS 2, *n* = 9). GPS, Glasgow Prognostic Score; ILD, interstitial lung disease; NSCLC, non‐small cell lung cancer; OS, overall survival. Median OS (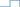) GPS 0: 16.9 months (*n* = 31), (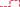) GPS 1: 9.8 months (*n* = 16), and (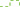) GPS 2: 7.6 months (*n* = 9).

The Kaplan–Meier survival curves for the patients are shown in Fig 2, with a one‐year survival of 46.1% and a median OS of 11.5 months. The individual group survival curves show the median OS for GPS 0 at 16.9 months, GPS 1 at 9.8 months, and GPS 2 at 7.6 months, which suggests that the GPS has significant prognostic value for NSCLC patients with ILD who undergo chemotherapy (*P* = 0.002) (Fig 3).

Univariate and multivariate analyses of the relationship between OS and each factor are shown in Table 3. Univariate analysis indicated an association between PS, GPS, and OS: hazard ratio (HR) of PS: 2.73, 95% CI: 1.16–6.37 (*P* = 0.020); HR of GPS 1: 2.59, 95% CI: 1.17–5.72 (*P* = 0.018); and HR of GPS 2: 5.27, 95% CI: 2.04–13.58 (*P* = 0.001). In multivariate analysis, only GPS 1 and GPS 2 were independent prognostic factors for OS in chemotherapy‐receiving NSCLC patients with pre‐existing ILD: HR of GPS 1: 2.34, 95% CI: 1.04–5.26 (*P* = 0.039) and HR of GPS 2: 4.61, 95% CI: 1.74–12.2 (*P* = 0.002). Although PS exhibited a trend toward association with OS, the results were not statistically significant (HR of PS: 1.82, 95% CI: 0.75–4.39 (*P* = 0.183).

**Table 3:** Univariate and multivariate analyses of overall survival in patients with lung cancer and ILD

	Univariate analysis			Multivariate analysis		
Variable	HR	95% CI	*P‐*value	HR	95% CI	*P*‐value
Age, per year increment	1.00	0.96–1.04	0.858			
Sex (male vs. female)	1.33	0.69–2.55	0.391			
Performance status (0, 1 vs. 2–4)	2.73	1.16–6.37	0.020	1.82	0.75–4.39	0.183
Smoking status (never vs. current or former)	1.95	0.46–8.19	0.361			
Pack‐years, per pack year increment	1.00	0.99–1.01	0.566			
BMI, per kg/m^2^ increment	0.90	0.81–1.01	0.084			
Histology (adenocarcinoma vs. nonadenocarcinoma)	0.87	0.47–1.63	0.677			
Clinical stage (III vs. IV or recurrence)	1.73	0.72–4.15	0.213			
WBC, per/μL increment	1.00	1.00–1.00	0.540			
Neutrophils, per/μL increment	1.00	1.00–1.00	0.526			
Lymphocytes, per/μL increment	1.00	0.99–1.00	0.988			
Eosinophils, per/μL increment	1.00	0.99–1.00	0.881			
KL‐6, per U/mL increment	1.00	0.99–1.00	0.564			
HRCT pattern (UIP pattern vs. non‐UIP pattern)	0.97	0.52–1.80	0.920			
Emphysema (yes vs. no)	1.02	0.49–2.12	0.938			
%predicted FVC, per % increment	0.98	0.97–1.00	0.157			
GPS						
0	1 (Ref)			1 (Ref)		
1	2.59	1.17–5.72	0.018	2.34	1.04–5.26	0.039
2	5.27	2.04–13.58	0.001	4.61	1.74–12.2	0.002

BMI, body mass index; CI, confidence interval; FVC, forced vital capacity; GPS, Glasgow Prognostic Score; HR, hazard ratio; HRCT, high‐resolution computed tomography; ILD, interstitial lung disease; KL‐6, Krebs von den Lungen‐6; Ref, reference; UIP, usual interstitial pneumonia; WBC, white blood cells.

## Discussion

GPS is a proven systemic inflammatory response‐based scoring system, measuring serum CRP and Alb levels.^19^, ^20^ It is a reliable prognostic indicator for patients with various forms of cancer including lung cancer.^19^, ^20^, ^21^, ^22^, ^23^ There have also been reports showing an association between GPS and chemotherapy‐related adverse events.^24^, ^33^ None of these studies, however, has focused on patients with presence or absence of concomitant ILD, AE‐ILD, or drug‐induced lung injury in their evaluations. To our knowledge, this is the first study to assess the GPS in this role, to show that it can predict the incidence of chemotherapy‐triggered AE‐ILD, and to demonstrate it as an independent prognostic tool of OS for chemotherapy‐receiving NSCLC patients with concomitant ILD.

Based on the 2016 International Working Group Report, AE‐IPF is categorized as an acute exacerbation that can be idiopathic or triggered (by infection, surgery, aspiration, or drug toxicity)^28^; chemotherapy‐related AE‐ILD is classified in the triggered category. Chemotherapy‐triggered AE‐ILD worsens the prognosis for lung cancer patients with concomitant ILD.^8^, ^11^ Predicting AE‐ILD is, therefore, necessary for effective treatment of lung cancer patients with concomitant ILD. Enomoto *et al*. ^29^ found low FVC as a risk factor for chemotherapy‐triggered AE‐ILD and Kenmotsu *et al*. ^34^ identified UIP pattern as a risk factor. However, our study found that the GPS, rather than FVC or UIP pattern, was a strong predictor for chemotherapy‐triggered AE‐ILD. The probable reasons for this result are: (i) we excluded gemcitabine due to the high risk of AE, but it was used in previous studies; and (ii) previous studies included patients with small cell lung cancer. Our study showed an ILD exacerbation rate of 9.6% within the first year of chemotherapy in patients with a GPS of 0, which was approximately the same as the annual exacerbation rate for IPF in patients without concomitant lung cancer (5%–15%).^35^ Conversely, patients with a GPS score of 2 exhibited a substantially higher exacerbation rate of 55.5% within the first year of chemotherapy. Therefore, we conclude that the GPS is a valuable predictive factor for chemotherapy‐triggered AE‐ILD in patients with lung cancer and pre‐existing ILD.

DOC‐triggered AE‐ILD occurred in 32% of the cases, the highest frequency among chemotherapy drugs (Table S2). Although DOC is key second‐line chemotherapy for NSCLC,^36^, ^37^ it may be avoided for lung cancer patients with simultaneous ILD because of the elevated risk of AE. In our study, the chemotherapy regimen was decided by each attending physician, which may have caused inconsistencies. However, this study excluded patients who underwent contraindicated regimens for ILD. The incidence of chemotherapy‐triggered AE‐ILD because of the potential impact of regimens was expected to be minimal.

There is a controversy about the involvement of inflammation in the chronic phase of ILD. In contrast, most reports suggest the involvement of inflammation in the acute phase of ILD.^25^, ^38^, ^39^, ^40^, ^41^, ^42^ For example, Song *et al*. ^39^ described high levels of circulating CRP as a prognostic factor for mortality of hospitalized patients with AE‐IPF. A high GPS level was identified as a prognostic factor for poor outcomes in patients with AE‐IPF.^25^ We found that the GPS, an indicator of inflammation, is associated with chemotherapy‐triggered AE‐ILD in lung cancer patients with concomitant ILD. Findings from our study confirm previous reports and suggest that inflammation plays an important role in AE‐ILD.

Alb is an indicator of the systemic nutritional status.^43^ According to Zisman *et al*.^44^ hypoalbuminemia is associated with a higher mortality rate among patients with ILD. Prealbumin, the precursor of Alb, has been recently reported to be a prognostic indicator in patients with IPF.^45^ In our study, we found that the GPS, which comprises Alb and CRP levels, predicts chemotherapy‐triggered AE‐ILD. This result supports the association between ILD and malnutrition. Low levels of serum Alb have been shown to increase susceptibility to the effects of inflammatory cytokines.^46^ It has also been reported that Alb has an antioxidant effect.^47^ These results suggest that hyperinflammatory states and unbalanced redox mechanisms associated with hypoalbuminemia powerfully trigger the cytotoxicity of chemotherapy and exacerbate ILD, worsening the outcomes.

Our study revealed an inverse relationship between the GPS and median OS rate in patients (Fig 3): as the GPS increases, the OS diminishes. The median OS was 16.9 months in patients with a GPS of 0, similar to lung cancer patients without ILD who received platinum‐based chemotherapy.^9^, ^48^, ^49^ The prognosis was worse (7.6 months) for patients with a GPS score of 2, probably because of the higher incidence of chemotherapy‐triggered AE‐ILD in these patients. Our study suggests that patients with NSCLC, pre‐existing ILD, and a higher GPS score are more likely to have an incidence of AE‐ILD. For these patients, we should take a more positive approach and observe progression according to the high incidence of AE‐ILD and poor OS. Currently, it is uncertain whether chemotherapy significantly prolongs OS compared with best supportive care for patients with lung cancer and concomitant ILD. Accordingly, we should probably consider avoiding chemotherapy as an alternative for lung cancer patients with ILD and high GPS. Conversely, patients with ILD, NSCLC, and a GPS score of 0 might have a better OS and benefit from a more aggressive chemotherapy regimen because of a lower risk for AE‐ILD. Furthermore, we calculated the GPS from blood test results before the onset of first‐line chemotherapy, according to the existing reports.^21^, ^22^, ^23^, ^24^ However, the GPS may change upon treatment or tumor activity. Recent studies have suggested that post‐treatment GPS can predict prognosis and treatment efficacy in patients with lung cancer.^50^, ^51^ Therefore, we may be able to predict a more accurate prognosis by focusing on GPS changes occurring during treatment.

It has been reported that the following six indicators can help predict the prognosis for patients with IPF: %predicted FVC, %predicted carbon monoxide diffusing capacity (DLCO), pulmonary hypertension, fibrosis score (degree of fibrosis and honeycomb lung in HRCT), modified Medical Research Council (mMRC) scale, and KL‐6.^27^, ^52^, ^53^, ^54^, ^55^ However, in the present study, %predicted FVC, UIP pattern in HRCT, and KL‐6 were not prognostic factors for all patients (Table 3). Moreover, for patients with routinely performed DLCO, %predicted DLCO also was not a prognostic factor (Table S3). Further larger‐scale studies are needed to determine whether ILD severity, activity, and comorbidity predict prognoses for lung cancer patients with ILD.

This study has some limitations. First, this was a nonrandomized retrospective study in a small number of patients. Different kind of biases and other confounding factors might be included in this study. However, to date, no large‐scale Phase 3 studies of pharmacotherapy have been conducted in NSCLC patients with ILD. Moreover, previous studies had sample sizes ranging from 21 to 114 patients,^1^, ^2^, ^3^, ^4^, ^5^, ^6^, ^7^, ^29^, ^30^, ^31^, ^32^, ^34^, ^38^ and many of them were as large as our study. Second, patients with ILD of a known etiology were excluded from this study because these diseases affect elevated CRP and/or hypoalbuminemia. Our results could have been influenced by this selection bias; and third, in our study, FVC values were higher than those in previous studies. The fact that patients with combined pulmonary fibrosis and emphysema were included in the study could have influenced our results. Fourth, we could not completely eliminate infection as a cause of AE‐ILD, and we did not perform bronchoalveolar lavage because it is a highly invasive procedure. Instead, we used blood cultures, sputum cultures, and other tests to rule out infection.

To conclude, the GPS is a valuable predictive tool for the risk of chemotherapy‐triggered AE‐ILD and a useful prognostic tool in lung cancer patients with pre‐existing ILD who receive chemotherapy. It is a simple and objective score calculated based on the serum CRP and Alb levels, and validation studies can be readily carried out. A prospective large‐scale multicenter study with a validation cohort is necessary to confirm the validity of our results.

## Disclosure

The authors declare there are no competing interests.

## Supporting information


**Table S1** Frequency of chemotherapy regimens during the clinical course.
**Table S2**. Incidence of chemotherapy triggered AE‐ILD in each chemotherapy.
**Table S3.** Univariate analysis of overall survival in patients who underwent carbon monoxide diffusing capacity.

**Figure S1.** Patient recruitment flow chart. NSCLC, non‐small cell lung cancer; ILD, interstitial lung disease; GPS, Glasgow Prognostic Score.
