# Supplementary material for: Glasgow Prognostic Score predicts chemotherapy‐triggered acute exacerbation‐interstitial lung disease in patients with non‐small cell lung cancer
Source: Thorac Cancer. 2021 Jan 21;12(5):667–75. doi: 10.1111/1759-7714.13792 (PMC7919129; doi:10.1111/1759-7714.13792)
Supplement: Supplementary file 1 — Table S1 Frequency of chemotherapy regimens during the clinical course. Table S2. Incidence of chemotherapy triggered AE‐ILD in each chemotherapy. Table S3. Univariate analysis of overall survival in patients who underwent carbon monoxide diffusing capacity. [file TCA-12-667-s001.pdf]

Glasgow Prognostic Score predicts chemotherapy-triggered acute exacerbation-interstitial lung disease in patients with non-small cell lung cancer

Ryota Kikuchi[a], Hiroyuki Takoi[a], Takao Tsuji[b], Yoko Nagatomo[a], Akane Tanaka[a], Hayato Kinoshita[a], Mariko Ono[a], Mayuko Ishiwari[a], Kazutoshi Toriyama[a], Yuta Kono[a], Yuki Togashi[a], Kazuhiro Yamaguchi[a], Akinobu Yoshimura[c], Shinji Abe[a,*]

Supplementary Table S1. Frequency of chemotherapy regimens during the clinical course

**First line (n = 56)**	**N**	**Second line (n = 35)**	**N**	**Third line (n = 13)**	**N**	**Fourth line (n = 4)**	**N**	**Fifth line (n = 2)**	**N**
CBDCA+PTX with/without BEV	29	DOC	19	DOC+RAM	3	DOC	1	S-1	1
CDDP/CBDCA+PEM with/without BEV	15	PEM	8	DOC	2	VNR	1	Nivo	1
CBDCA+Nab-PTX	6	S-1	4	PEM	2	Nab-PTX	1		
CBDCA+S1	3	CBDCA+ PTX	1	Nivo	2	Nivo	1		
CDDP/CBDCA+VNR	2	VNR	1	VNR	1				
CDDP+ETP	1	Nivo	1	Nab-PTX	1				
		Pembrolizumab	1	S-1	1				
				Atezolizumab	1				

N, number; CBDCA, carboplatin; PTX, paclitaxel; BEV, bevacizumab; DOC, docetaxel; RAM, ramucirumab; CDDP, cisplatin; PEM, pemetrexed; VNR, vinorelbine; Nivo, nivolumab; Nab-PTX, nanoparticle albumin-bound paclitaxel; ETP, etoposide.

Supplementary Table S2. Incidence of chemotherapy triggered AE-ILD in each chemotherapy regimen

Chemotherapy	Incidence of AE
Platinum agents (n = 57)	4 (7.0%)
PTX (n = 30)	1 (3.3%)
DOC (n = 25)	8 (32%)
PEM (n = 25)	3 (12%)
S-1 (n = 9)	0 (0%)
Nab-PTX (n = 8)	0 (0%)
VNR (n = 6)	0 (0%)
Others (n = 8)	0 (0%)

AE, acute exacerbation; ILD, interstitial lung disease; PTX, paclitaxel; DOC, docetaxel; PEM, pemetrexed; Nab-PTX, nanoparticle albumin-bound paclitaxel; VNR, vinorelbine; ETP, etoposide. Others: Nivolumab (n = 5), Pembrolizumab (n = 1), Atezolizumab (n = 1), ETP (n = 1)

Supplementary Table S3. Univariate analysis of overall survival in patients who underwent carbon monoxide diffusing capacity (n = 21)

**Variable**	**Univariate analysis**		
	HR	95% CI	*p* value
**%predicted DLCO,** per %increment	1.016	0.99–1.03	0.155

HR, hazard ratio; CI, Confidence interval; DLCO, carbon monoxide diffusing capacity.

Supplementary Fig. S1. Patient recruitment flow chart. NSCLC, non-small cell lung cancer; ILD, interstitial lung disease; GPS, Glasgow Prognostic Score.

Supplementary Table S1. Frequency of chemotherapy regimens during the clinical course.

Supplementary Table S2. Incidence of chemotherapy triggered AE-ILD in each chemotherapy.

Supplementary Table S3. Univariate analysis of overall survival in patients who underwent carbon monoxide diffusing capacity.
