# Supplementary figures and images for: Glasgow Prognostic Score predicts chemotherapy‐triggered acute exacerbation‐interstitial lung disease in patients with non‐small cell lung cancer
Source: Thorac Cancer. 2021 Jan 21;12(5):667–75. doi: 10.1111/1759-7714.13792 (PMC7919129; doi:10.1111/1759-7714.13792)

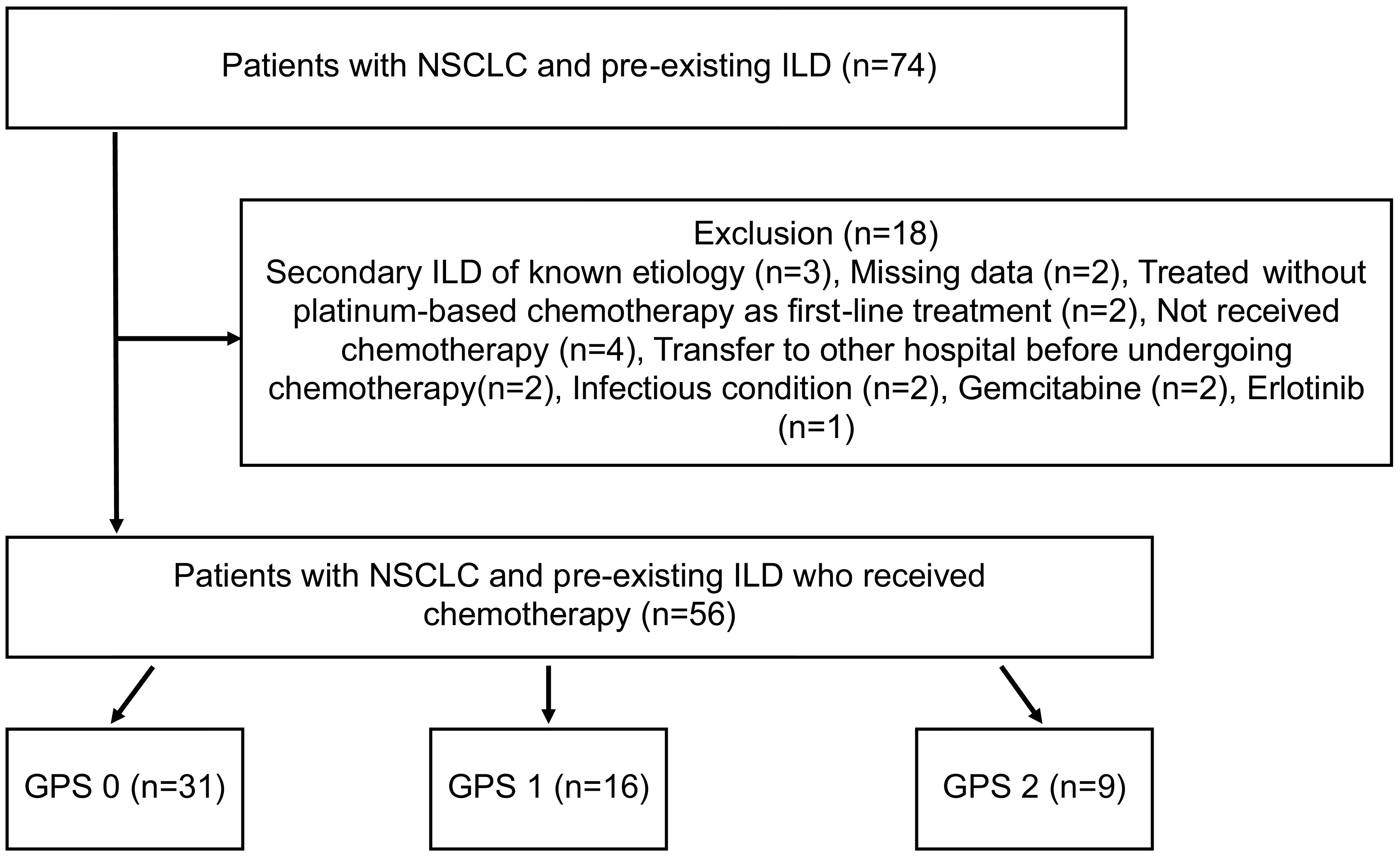

Supplement: Supplementary file 2 — Figure S1. Patient recruitment flow chart. NSCLC, non‐small cell lung cancer; ILD, interstitial lung disease; GPS, Glasgow Prognostic Score. [file TCA-12-667-s002.tif]
